# World netball cardiac screening guidelines

**DOI:** 10.17159/2078-516X/2022/v34i1a13979

**Published:** 2022-01-01

**Authors:** L Bogwasi, DC Janse van Rensburg, G Bryant, J Orchard, J A Drezner

**Affiliations:** 1 Section Sports Medicine, Faculty of Health Sciences, University of Pretoria, South Africa; 2 Nyangabgwe Hospital, Orthopedic Department, Francistown, Botswana; 3 Medical Member, Confederation of African Football, Cairo, Egypt; 4 World Netball Medical Commission, Manchester, UK; 5 Sports Medicine at Sydney University, The Sports Clinic, The University of Sydney; 6 Centenary Institute, The University of Sydney, Sydney, Australia; 7 Center for Sports Cardiology, University of Washington, Seattle, WA, USA

**Keywords:** sudden cardiac death, sudden cardiac arrest, pre-participatory medical assessment

## Abstract

Sudden cardiac adverse events remain an area of concern in sport. The precise risk for netball athletes is unknown but the annual incidence of sudden cardiac death in sports is reported at 0.5–2 cases in 100 000 young competitive athletes between the ages of 12–35 years. Cardiac screening in the sport and exercise medicine context aims at identifying pathologies associated with catastrophic events when combined with physical activity. There is an ongoing debate relating to the standardisation of the pre-participatory medical assessment (PPMA). World Netball (WN) commissioned a cardiac screening policy (13 March 2022). The minimum PPMA recommended by World Netball is a history, physical examination, and a resting 12-lead electrocardiogram (ECG). ECGs should be interpreted in accordance with athlete-specific ECG interpretation criteria. Expansion of sports cardiology experience and infrastructure, in combination with universal emergency response planning for sudden cardiac arrest, is intended to safeguard athlete health and player welfare in WN.

The health benefits of physical activity are well documented.^1^–^2^ It is known, however, that intense physical activity in the setting of underlying cardiac pathology can trigger potential catastrophic cardiac events, such as sudden cardiac arrest or death during sport.^3^

These cardiac events have been recorded worldwide in different sporting codes at both amateur and elite levels.^3^ It has led to more emphasis being placed on the pre-participation medical assessment (PPMA) as a recommended practice for athletes before engaging in physical activity.^3^ The PPMA is conducted in the pre-season or before any major competition at regional, national, and international levels.^4^–^6^ Netball forms part of this cohort of exertional physical activities and as such, a World Netball cardiac screening policy is of great importance. The contents of a PPMA may vary based on resource availability, sports medical expertise, discretion or availability and, most importantly, the recommendation of the particular sport regulatory body.

Although cardiac screening is not one-hundred percent effective in preventing cardiac incidents during sporting activity,^7^ it aims at identifying those pathologies associated with catastrophic events when combined with physical activity. Cardiac screening has been an area of focus within the sport and exercise medicine community to curb morbidity and/or mortality from sudden cardiac arrest in competitive athletes. Other consequences from catastrophic incidents include the psychological trauma placed on the athletes’ family, team members, spectators, and community, along with the magnitude of attention it carries towards the sporting code, team, national and international federations and the team physicians. Although tragic events are rare, they have a huge impact.^8^

Sudden cardiac arrest is the cause of 75% of deaths during sport. 9 Although the precise risk in netball athletes is unknown, the incidence of sudden cardiac death in sports is reported at 0.5–2 cases in 100 000 in young competitive athletes between ages 12–35 years.^3^,^10^ With proper emergency planning, a significant proportion of the victims survive. 8 However, the 20–33% that do not survive despite immediate resuscitation with an Automated External Defibrillator (AED),^11^–^13^ reiterates that prevention is better than cure.

Early identification of detectable pathologies is very important regardless of the low prevalence (0.3%) of cardiac abnormalities associated with sudden cardiac arrest or death. 14 There is a heterogeneity of causes, such as cardiomyopathies, long QT syndrome, idiopathic left ventricular hypertrophy, myocarditis and anomalous coronary arteries. Hypertrophic cardiomyopathy is the most common of these and accounts for 8–36% of cardiac pathologies identified after sudden cardiac death in the athlete population.^10^,^15^ In up to 44% of athletes that suffered sudden cardiac death, no structural abnormalities are seen at post-mortem, with a proportion thought to be caused by primary electrical disorders.^16^ There is ongoing debate regarding standardisation or individualisation of cardiac screening tests in athlete populations concerning age, sex, family history, race, sport, and level of activity, weighed against the risks associated with the potential interventions for the cardiac conditions detected.^15^

## Cardiac screening in physical activity

The basics of a cardiac screening programme entail a detailed history, physical examination and an electrocardiogram (ECG) with further tests such as an echocardiogram, cardiac magnetic resonance imaging (cMRI), ambulatory or stress ECG when baseline results are unclear or abnormal.

There is a continuing debate on the mandatory inclusion of a 12-lead ECG in a routine pre-participatory screening with scrutiny regarding its detection rate, availability, and the ability of the physician to provide an athlete-specific accurate interpretation. In athlete populations, the ECG detects 60% of cardiac pathologies at risk of sudden death with a low false-positive rate (1.3%) when interpreted by experienced physicians.^17^ When a standardised athlete-specific ECG interpretation is applied, the sensitivity and specificity of the ECG in detecting cardiac abnormalities is improved.^18^,^19^ When contemporary athlete-specific ECG standards are used by clinicians with ECG interpretation experience, approximately one in six abnormal ECGs (positive predictive value) will represent a pathologic cardiac disorder associated with sudden cardiac arrest and death.^17^

There are limitations in detecting cardiac pathologies when using only patient history and physical examination, as up to 80% of athletes with underlying cardiac disorders may not display symptoms.^7^,^20^,^21^ Augmentation of the PPMA with an ECG is therefore important and should be done whenever accessible by physicians capable of accurate ECG interpretation with access to cardiology resources for secondary testing of ECG abnormalities. An ECG with an abnormal finding or more than one borderline finding according to the International Criteria for athlete ECG interpretation^22^ guides further cardiac evaluation.

## Evidence-based recommendations

The best available evidence suggests that cardiac screening does not eliminate all risk of cardiac-associated adverse events in physical activity. However, the detectable cases from cardiac screening are important to promote an environment conducive to the safe participation of the athlete.^23^ Based on the current evidence, World Netball through its medical committee developed and adopted these cardiac screening guidelines with support from two independent cardiac experts. The minimum PPMA recommended by World Netball is a history, physical examination and a resting 12-lead ECG. ECGs should be interpreted in accordance with athlete-specific ECG interpretation criteria (currently the International Criteria).^22^

## Guidelines for member associations

### 1. National level

- Cardiac screening is recommended for all netball players aged 16 years and older. Netball players that are younger than 18 years must undergo cardiac screening in the presence of an adult guardian. Cardiac screening in athletes older than 35 years should shift focus to a risk assessment for atherosclerotic cardiovascular disease.
- Netball players should undergo cardiac screening at least three to four weeks before the start of the season, or the first major competition.
- Netball players should have cardiac screening annually, or at least every two years, guided by the availability of resources.
- Netball players with positive test results during screening need further evaluation. Some players with screening abnormalities will be advised to pause playing and training until the secondary evaluation is complete. For players who have been recommended to stop playing and training due to an abnormality found on cardiac screening, the player is not to return until cleared by a specialist cardiologist. When cleared to play, the player must be followed up at the interval recommended by their cardiologist.
- Any player who develops cardiac symptoms during the season should seek medical evaluation and follow-up as required.
- Member associations are encouraged to have close contact with a qualified sports medicine physician for cardiac screenings.
- Member associations are encouraged to develop a cardiology infrastructure, including a referral network of cardiologists (or sports cardiologists), for evaluation of screening abnormalities and participation guidance if a cardiac disorder is identified.
- Member associations are encouraged to have recurring educational training regarding cardiac issues in sports, including the role and limitations of cardiac screening, and the recognition and management of sudden cardiac arrest on the field-of-play.
- National associations must have a clear cardiac resuscitation plan with qualified responders and a functional AED (please write out in full when used for the first time) available before any netball game or national team training. This procedure is also encouraged at regular team training.
- Sudden cardiac arrest should be assumed in any athlete that collapses and is unresponsive. Immediate resuscitation measures include: 1) activating the emergency medical response system, 2) cardiopulmonary resuscitation starting with chest compressions, and 3) retrieval and application of the AED as soon as possible.
- Interruptions in chest compressions should be minimised and AED first shock time should be less than 3 minutes after collapse. Emergency preparedness also extends to the transfer to in-hospital care and post-cardiac event care and rehabilitation.
- Member associations must implement reporting of sudden cardiac arrest and death in sports as a recordable event and develop a clear chain of reporting to World Netball through the national association. In cases of sudden death, a post-mortem is encouraged through the consent of the guardians where possible.

### 2. Team physicians

- Must conduct PPMA of the team during the pre-season or before the first major competition.
- Are encouraged to provide players with information on the benefits and limitations of cardiac screening.
- Are encouraged to have a background in athlete cardiac screening and, in particular, have undergone training on ECG interpretation in athletes.^24^ Free online training modules for the International Criteria are available at: https://uwsportscardiology.org/e-academy/
- Should form a working relationship with a local cardiologist for organising follow-up if required and management of complex cases.
- Must educate their athletes on cardiac issues in sports and encourage them to report symptoms.
- Are encouraged to have a good working relationship with their players allowing them to openly report symptoms without fear.
- Are responsible to develop an emergency action plan for sudden cardiac arrest with the appropriate education of their coaches and staff.

### 3. Players

- Are encouraged to be the custodians of their health and give correct answers during screening evaluations and report symptoms.

### 4. Coaches

- Are encouraged to fully support their medical team and be trained in cardiopulmonary resuscitation (CPR) and the use of an AED.
- Must have a good relationship allowing players to confide in them without fear.

**Fig. 1:**
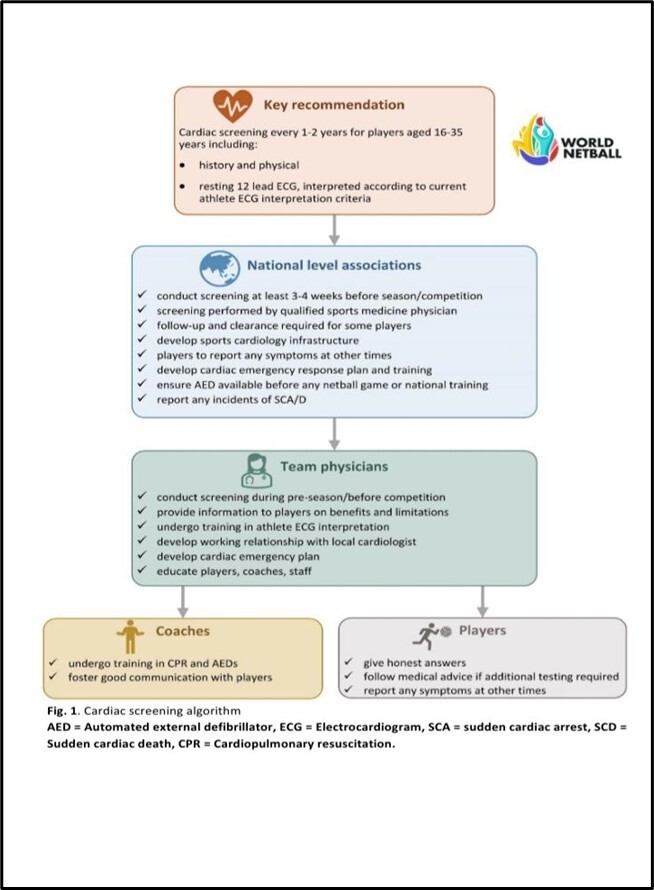
Cardiac screening algorithm. AED, automated external defibrillator; ECG, electrocardiogram; SCS, sudden cardiac arrest; SCD, sudden cardiac death; CPR, cardiopulmonary resuscitation

Figure 1 summarises the recommendations of the World Netball Cardiac Screening Policy.

## Conclusion

Medical professionals and teams must ensure cardiac screening of their athletes. Although this does not eliminate the risks of cardiac-related adverse events, it ensures that detectable cases from cardiac screening are worked up and managed accordingly. While this cardiac screening policy was written explicitly for World Netball, the guidelines apply to any sport to promote an environment conducive to the safe participation of the athlete.
